# Center of Pressure Analysis of Postural Stability During Repetitive Reaching with Passive Arm-Support Exoskeletons

**DOI:** 10.3390/s25185650

**Published:** 2025-09-10

**Authors:** Byungkyu Choi, Jaehyun Park

**Affiliations:** Department of Industrial Engineering, Konkuk University, Seoul 05029, Republic of Korea; bkchoi@konkuk.ac.kr

**Keywords:** stabilometry, force plate analytics, wearable robotics, amplitude probability density function (APDF), ergonomics

## Abstract

This study assessed the effects of passive arm-support exoskeletons (ASEs) on postural stability during repetitive arm-reaching tasks. In a 3 × 3 × 2 within-subject design, twenty-four healthy right-handed men completed left-, front-, and right-facing arm-reaching tasks at two working distances (65.5 and 68.9 cm) under three intervention conditions (Without, VEX, Airframe). Postural stability was assessed using center of pressure (CoP) data recorded from a force plate. Both ASEs clearly reduced the mean amplitude of CoP in the mediolateral (ML) direction (i.e., the absolute value of MEAN ML and ML APDF10), although neither yielded improvements in anteroposterior (AP) stability. Task direction significantly influenced all CoP measures: left-facing tasks produced the greatest leftward bias, whereas front-facing tasks yielded the smallest AP CoP amplitude. Increasing the working distance by <4 cm modestly heightened AP bias, as reflected in larger AP bias metrics (i.e., MEAN AP, ML APDF50, and ML APDF90). Overall, passive ASEs selectively enhanced lateral postural control, while their effect on AP stability was negligible or even slightly adverse. These findings indicate that the practical utility of passive ASEs depends on the directional demands of specific occupational tasks.

## 1. Introduction

Repetitive arm-reaching motions are fundamental upper-limb movements commonly performed in various occupational and daily contexts. Although typically low in intensity, the physical demands can increase significantly when these movements involve repetitive cycles, asymmetric postures, or changes in direction, as they place greater demands on postural control and trunk stability [1,2]. If such demands persist, they can lead to fatigue or discomfort, potentially compromising both postural stability and task efficiency. Consequently, maintaining proper posture and balance during repetitive arm movements should be recognized as critical ergonomic considerations in task design.

Arm-support exoskeletons (ASEs) are wearable devices designed to offload the weight of the upper limbs, thereby reducing muscular demand and supporting posture maintenance during arm-elevating tasks [3,4,5,6,7]. Previous studies have predominantly focused on the ability of ASEs to lower electromyographic (EMG) activity and improve work efficiency in repetitive overhead tasks [8,9,10,11,12,13]. More recently, however, the scope of ASE applications has expanded, with growing interest in whether these devices also influence users’ postural control and balance regulation strategies—beyond their intended function of supporting the upper limbs [14,15,16].

Of course, in lightweight, repetitive tasks such as simple arm-reaching motions, the use of an exoskeleton may not be strictly necessary. In fact, under such conditions, ASEs may inadvertently restrict trunk mobility or interfere with balance control. This raises important questions about how these devices interact with the body’s overall postural regulation mechanisms—beyond merely assisting arm movement.

Postural stability can be quantitatively assessed through center of pressure (CoP) analysis, a widely used metric that reflects an individual’s ability to maintain balance. Prior studies have reported mixed findings regarding the influence of exoskeleton use on CoP behavior. Maurice et al. [9] found that ASE use reduced CoP displacement, suggesting improved postural stability. In contrast, a study by Park et al. [17] reported that back-support exoskeleton (BSE) use increased CoP sway due to movement constraints, potentially undermining balance control. Although the device types differ, these conflicting findings suggest that the effects of exoskeletons on postural stability may vary depending on their design characteristics, wearing conditions, and usage context.

Accordingly, this study aimed to quantitatively investigate the impact of ASE use on postural stability during a lightweight but highly repetitive task simulating arm-reaching movements in a controlled laboratory setting. Unlike most prior studies that have primarily examined overhead or load-intensive tasks with a focus on electromyographic responses, this study contributes new insights by employing force plate-based CoP analysis to capture balance-related effects of ASEs. Furthermore, by systematically varying task direction and working distance, the present work highlights how even subtle spatial factors shape the stability outcomes of exoskeleton use. This approach provides a novel perspective on the practical utility and limitations of passive ASEs in everyday repetitive reaching activities, beyond the traditionally studied overhead scenarios.

## 2. Methods

### 2.1. Participants

Twenty-four healthy right-handed adult men voluntarily participated in this study. The mean ± standard-deviation (SD) age, height, and body weight were 24.1 ± 1.9 years, 176.0 ± 4.6 cm, and 73.9 ± 8.1 kg, respectively. All participants performed the experimental tasks using their dominant (right) hand.

### 2.2. Exoskeletons Evaluated

This study evaluated two types of passive ASEs: VEX (Hyundai-Rotem Co., Uiwang-si, Republic of Korea) and Airframe (Levitate Technologies Inc., San Diego, CA, USA). The VEX operates via a multi-link mechanism and is designed to assist arm elevation within a shoulder flexion range of approximately 100–130° from the neutral posture. It weighs approximately 2.8 kg and provides five selectable levels of torque assistance ranging from 3.8 to 8.6 Nm; for this study, the assistance level was fixed at 4.7 Nm. The Airframe is a spring-based passive exoskeleton weighing approximately 3.2 kg. In this study, it was configured to provide a higher support torque of 7.5 Nm compared with the VEX. Both devices were worn like a backpack and allowed individual adjustments of shoulder and waist straps, as well as frame length, to accommodate different user body dimensions. The assistance levels (VEX: 4.7 Nm, Airframe: 7.5 Nm) were selected based on prior ergonomic evaluations, where assistive forces in this range were found effective without overly limiting natural adjustments [9,14,18].

### 2.3. Experimental Task and Design

**Figure 1:**
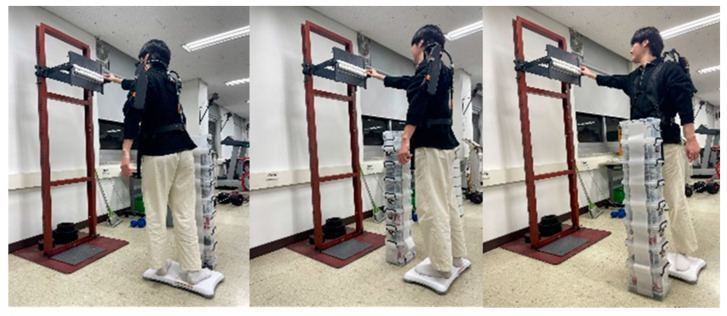
Examples of the experimental tasks (left-facing, front-facing, and right-facing, shown left to right).

This study was designed to quantitatively assess the effects of ASE use on postural stability during simulated repetitive upper-limb tasks in a laboratory setting. Participants performed the task while standing naturally with both feet shoulder-width apart on a force plate for center of pressure (CoP) measurement. The midpoint between their feet was aligned with the center of the plate to ensure consistent positioning. Each participant was instructed to remove 20 Styrofoam balls (40 mm in diameter, weighing approximately 2 g each) affixed to a task board at a height of 175 cm and transfer them, one by one, into a collection box positioned 100 cm in front of them using the right hand (Figure 1), following the procedure by Choi and Park [19]. This task was designed to involve repetitive arm lifting and lowering, elbow flexion and extension, shoulder abduction and adduction, and partial trunk rotation depending on task direction—thereby incorporating a range of upper-body movement conditions.

This study employed a 3 × 3 × 2 within-subject design with three independent variables: intervention condition (Without, VEX, Airframe), task direction (left-facing −90°, front-facing 0°, right-facing 90°), and working distance (near 65.5 cm, far 68.9 cm). The experimental task was performed under 18 distinct conditions created by all possible combinations of these variables. Task direction was defined by the location of the task board relative to the participant, who maintained a forward-facing posture throughout all conditions and performed the task at the left, front, or right position accordingly. Working distances were determined from the 50th and 75th percentiles of combined arm-plus-palm length for Korean adults, calculated from the 8th Korean Anthropometric Survey [20], and set at 65.5 cm and 68.9 cm, respectively.

**Figure 2:**
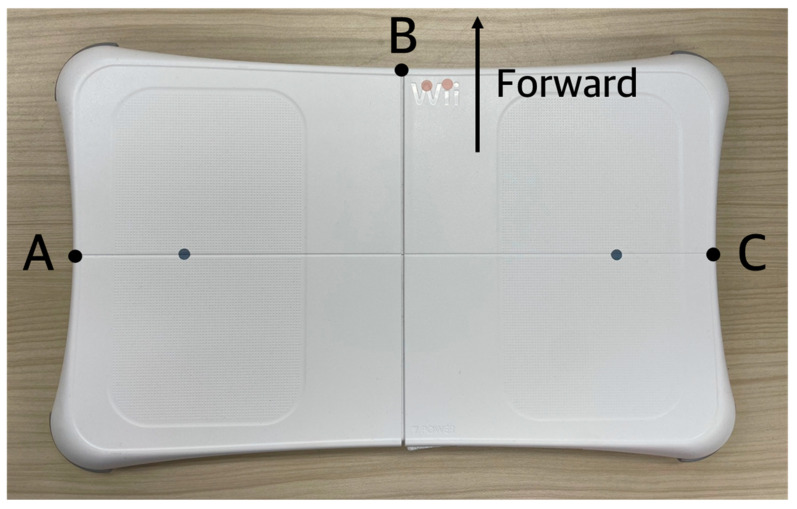
Reference points A, B, and C on the force plate, used to set working positions for left-, front-, and right-facing tasks, respectively. For example, during the far working distance in left-facing tasks, point A was positioned approximately 68.9 cm from the vertical line passing through the center of the task surface.

To maintain consistent working distances across all experimental conditions, the force plate was positioned so that specific reference points on its surface corresponded precisely to the predetermined distances of 65.5 cm and 68.9 cm from the task surface (Figure 2). For left- and right-facing tasks, the left and right endpoints of the force plate’s horizontal centerline were aligned with the designated working distances. For the front-facing task, the front-most point of the force plate served as the reference. These defined reference points functioned as stable spatial markers, ensuring the reliable and reproducible placement of the force plate in accordance with anthropometric standards.

To ensure a balanced presentation of conditions and control for order effects, a Latin square design was employed. For the two factors with three levels—intervention condition and task direction—a 3 × 3 balanced Latin square was applied. For working distance, which had two levels (near and far), a separate 2 × 2 Latin square was implemented. This approach allowed the 18 combinations of experimental conditions to be evenly distributed across participants while minimizing potential sequence effects.

This experimental framework was selected to systematically examine not only the main effects of ASE use, but also the potential interaction effects with spatial task characteristics. Task direction was included because asymmetric reaching across the body midline can impose distinct balance demands compared with front-facing tasks. Working distance was varied to test whether even small extensions within the normal reach envelope could alter postural control. Together, this design allowed us to explore how ASE support interacts with common spatial factors that are relevant to many occupational tasks.

### 2.4. Experimental Procedure

Prior to the experiment, all participants were verbally informed of the study’s purpose, procedures, experimental tasks, and potential risks. They were also clearly advised of their right to withdraw from the study at any time without penalty. After providing written informed consent, participants completed a demographic questionnaire. Both ASEs were then individually adjusted to fit each participant’s body dimensions. A familiarization session was provided to allow participants sufficient time to adapt to the equipment and practice the task procedures.

Each participant completed all 18 experimental conditions, representing every combination of the three experimental factors: intervention condition, task direction, and working distance. To minimize cumulative fatigue, a three-minute rest period was provided between conditions.

This study was approved by the Institutional Review Board of Incheon National University.

### 2.5. CoP Measurement

To assess postural stability, CoP data were obtained using the Wii Balance Board (Nintendo Co., Ltd., Kyoto, Japan), which has been validated as a reliable and research-grade instrument for assessing static postural control in laboratory-based studies [21,22,23]. Data were recorded at a sampling rate of 100 Hz using BrainBLox (Neuromechanics Laboratory, University of Colorado Boulder, CO, USA).

To quantitatively evaluate postural stability, CoP signals were analyzed using both mean-based and probability-based metrics. The mean-based indicators included medial–lateral CoP displacement (MEAN ML) and anterior–posterior CoP displacement (MEAN AP). These variables represent the mean amplitude of CoP and were calculated as the average of all time-series data points collected throughout the trial, specifically the mediolateral (ML) and anteroposterior (AP) coordinates measured in centimeters.

MEAN ML and MEAN AP thus represent the average displacement of the CoP along the ML and AP axes, respectively. These arithmetic means are widely used to assess positional bias and directional tendencies in posture, while also serving as representative measures of postural stability [24]. When these values consistently deviate in a specific direction, they can provide insight into systematic directional bias during balance control. In particular, when the standard deviation is small—indicating low variability—the mean value itself can be interpreted as a reliable indicator of directional tendency. Conversely, a large standard deviation suggests inconsistent sway direction, even if the mean is small, and thus warrants more cautious interpretation.

The overall mean amplitude of CoP, reflecting the extent of total body sway, is considered one of the most reliable indicators of postural control ability. In general, a higher amplitude indicates poorer balance control, whereas a lower value suggests greater stability. This measure has demonstrated high test–retest reliability (intraclass correlation coefficient, ICC = 0.70–0.90) [25], especially when foot positioning is standardized across trials [24,26]. Additionally, averaging data across multiple trials helps minimize random noise and improves the robustness of the analysis [24].

To more sensitively capture the distributional characteristics of postural sway, the amplitude probability density function (APDF) analysis was employed as a complementary probability-based metric. Although APDF has primarily been used to interpret the amplitude distribution of electromyographic (EMG) signals in a cumulative probability format, its application was tentatively extended to CoP signals in the present study. Because CoP data also comprise time-series displacement amplitudes, this analytical approach is conceptually appropriate. Notably, APDF offers quantitative insight into the magnitude and distributional properties of sway that are not easily captured by simple mean or standard-deviation measures, making it potentially useful for detecting subtle differences in postural stability.

In this study, APDF values at the 10th, 50th, and 90th percentiles were calculated separately for the ML and AP axes of CoP. APDF10 represents the amplitude below which 10 % of the sway data are distributed during the measurement period; a higher value indicates that even the smaller fluctuations occurred with greater magnitude. APDF50 reflects the median sway amplitude and thus the typical sway intensity, whereas APDF90 captures the magnitude of the largest, less-frequent deviations. Overall, higher APDF values suggest larger or more frequent postural fluctuations, which may be interpreted as indicative of reduced postural stability. This interpretation is conceptually aligned with the analytical framework originally proposed for EMG data, in which APDF10, APDF50, and APDF90 represent static, average, and peak load levels, respectively [27].

### 2.6. Data Analysis

The dependent variables for postural stability analysis included MEAN ML and MEAN AP, as well as the APDF metrics APDF10, APDF50, and APDF90 for both ML and AP axes. CoP data were postprocessed using MATLAB R2024a (MathWorks Inc., Natick, MA, USA). An RMS filter with a 100 ms moving window was applied, and APDF values were subsequently computed for both axes.

Although the Shapiro–Wilk test indicated deviations from normality for some variables, the absolute skewness and kurtosis values for all distributions were within widely accepted thresholds (|skewness| < 2, |kurtosis| < 7), supporting the use of parametric inference [28,29]. Homogeneity of variances was also confirmed by Levene’s test. In line with prior evidence that MANOVA is generally robust to moderate violations of normality in balanced within-subject designs [30,31], parametric testing was considered appropriate.

To further reinforce the validity of this choice, the findings were also cross-checked using robust alternatives commonly recommended for non-normal data, including non-parametric and permutation-based MANOVA approaches [32]. The results of these supplementary analyses were consistent with those from the parametric MANOVA, indicating that the significant main and interaction effects observed in this study were not driven by violations of distributional assumptions. This convergence across methods strengthens confidence in the reliability of the reported outcomes.

Accordingly, MANOVA was used to examine the effects of intervention condition, task direction, and working distance on postural stability. When significant main or interaction effects were observed, post hoc comparisons were conducted using the Student–Newman–Keuls (SNK) test. All statistical analyses were performed with IBM SPSS Statistics version 28 (IBM Corp., Armonk, NY, USA), using a significance level of α = 0.05.

## 3. Results

### 3.1. Interaction Effects

**Figure 3:**
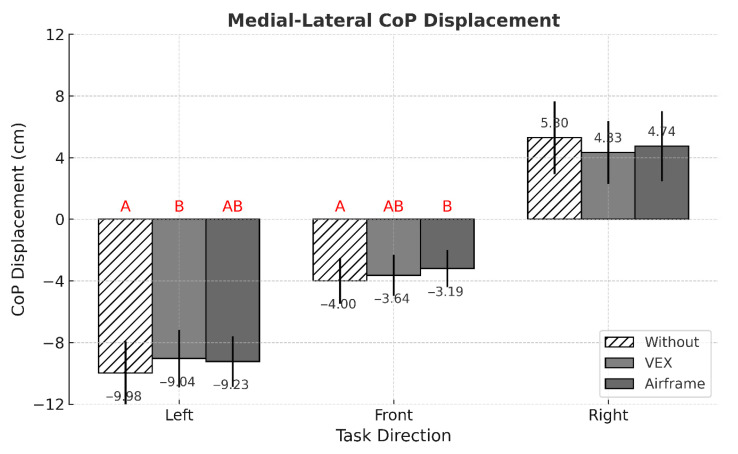
Mean comparison of MEAN ML across intervention conditions and task directions (*p* = 0.004, η^2^_p_ = 0.037). Error bars represent standard deviations. Different letters above the bars denote statistically significant differences among intervention conditions within each task direction based on simple main effects analysis (*p* < 0.05).

**Figure 4:**
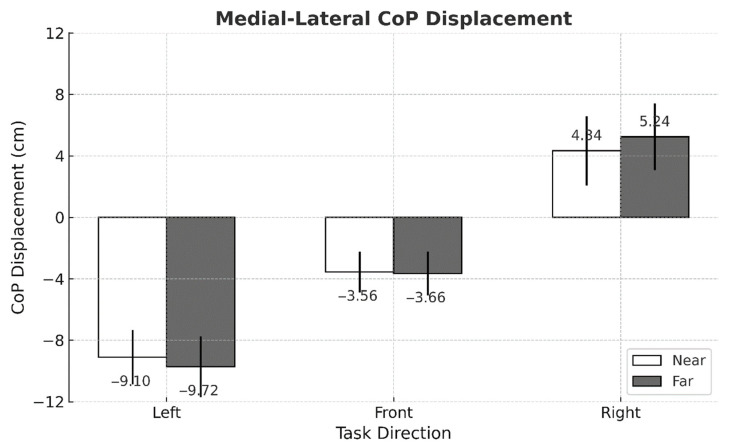
Mean comparison of MEAN ML across task directions and working distances (*p* = 0.002, η^2^_p_ = 0.030). Error bars represent standard deviations.

**Figure 5:**
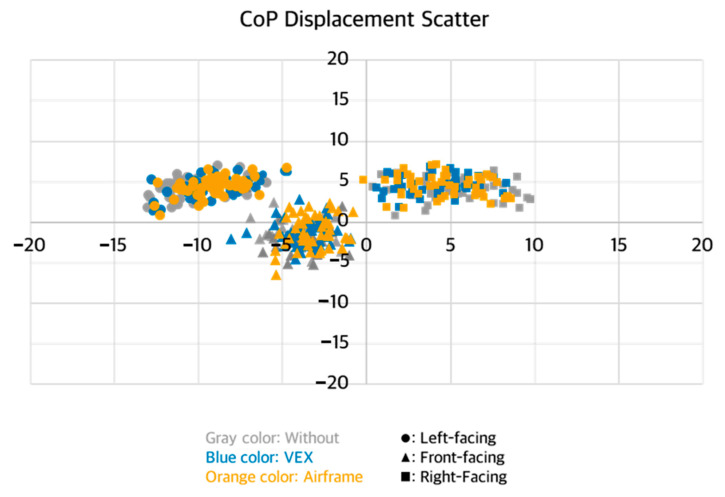
Scatter plot of mean CoP positions by intervention condition and task direction.

No significant three-way interaction among intervention condition, task direction, and working distance was detected for any postural stability indicator (Table 1). By contrast, MEAN ML exhibited two significant two-way interactions—intervention × task direction (*p* = 0.004) and task direction × working distance (*p* = 0.002) (Figure 3 and Figure 4). Across all task directions, ASE use consistently lowered the absolute value of MEAN ML, whereas performing the task at the far working distance consistently elevated it (Figure 5).

**Figure 6:**
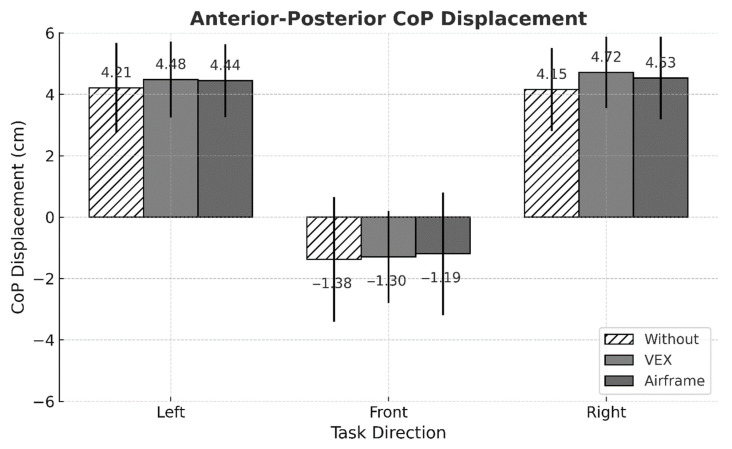
Mean comparison of MEAN AP across intervention conditions and task directions (*p* > 0.05, not significant). Error bars represent standard deviations.

MEAN AP showed no statistically significant interaction effects. Nevertheless, descriptive inspection revealed that the absolute value of MEAN AP tended to increase under ASE use during left- and right-facing tasks (Figure 5 and Figure 6).

**Figure 7:**
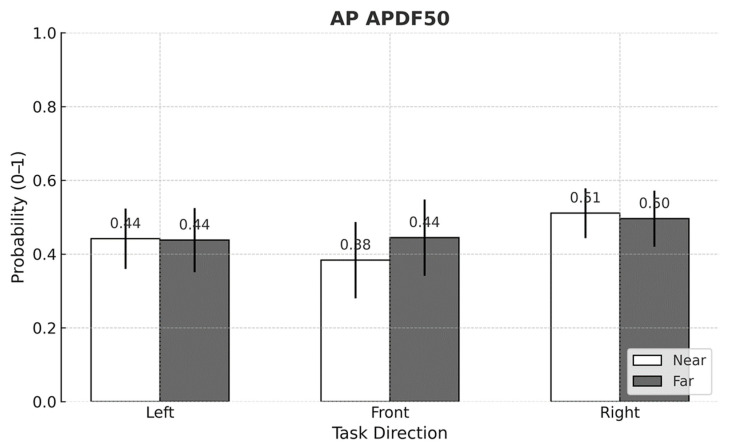
Mean comparison of AP APDF50 across task directions and working distances (*p* < 0.001 η^2^_p_ = 0.036). Error bars represent standard deviations.

**Figure 8:**
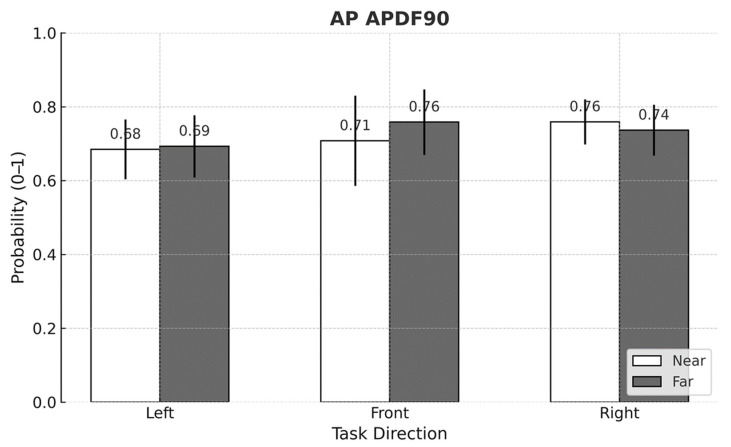
Mean comparison of AP APDF90 across task directions and working distances (*p* = 0.002, η^2^_p_ = 0.030). Error bars represent standard deviations.

For APDF metrics, significant task direction × working distance interactions were found for AP APDF50 (*p* < 0.001) and AP APDF90 (*p* = 0.002) (Figure 7 and Figure 8). Aside from the front-facing condition, neither metric demonstrated a consistent pattern across task directions and distances. Within the front-facing task, both AP APDF50 and AP APDF90 rose by up to 15.8% when the working distance increased from near to far.

### 3.2. Intervention

ML APDF10 values were significantly lower under both ASE conditions than under the Without condition (*p* = 0.003) (Table 2). No other postural stability variables differed significantly among intervention conditions (*p* > 0.05). Non-significant trends were observed: ML APDF50 and ML APDF90 tended to decrease, whereas AP APDF10, APDF50, and APDF90 tended to increase with ASE use.

### 3.3. Task Direction

Statistically significant differences across all CoP indicators were found among task directions (Table 3). The absolute value of MEAN ML was greatest during left-facing tasks, followed by right-facing and front-facing tasks. Conversely, the absolute value of MEAN AP was lowest during front-facing tasks. Among the APDF metrics, ML APDF values were highest during left-facing tasks, whereas AP APDF values were highest during right-facing tasks.

### 3.4. Working Distance

A significant main effect of working distance was found for MEAN AP (*p* = 0.001): the mean value was lower at the far working distance than at the near working distance, although this difference was accompanied by large standard deviations (Table 4). Working distance also significantly affected ML APDF50 (*p* = 0.010) and ML APDF90 (*p* = 0.020), with both metrics showing higher values under the far-distance condition.

## 4. Discussion

This study set out to determine whether using passive arm-support exoskeletons (ASEs) alters postural stability during a lightweight yet highly repetitive arm-reaching task performed in different directions and at two working distances. Three principal observations emerged. First, ASE use consistently attenuated mediolateral (ML) sway, as evidenced by lower MEAN ML amplitudes and reduced fine-scale variability (ML APDF10). This outcome may be attributed to the general unloading effect of ASEs, which can reduce lateral demands on postural control. Second, task direction exerted a dominant influence on center of pressure (CoP) behavior: left-facing trials produced the greatest leftward bias and ML variability, whereas front-facing trials showed the smallest anteroposterior (AP) deviations. Third, although the difference between the two working distances was modest (<4 cm), extending the reach distance modestly increased ML APDF50 and ML APDF90 while decreasing MEAN AP, indicating distance-dependent adjustments in postural control. Notably, MEAN ML responded to two distinct two-way interactions—intervention × direction and direction × distance—underscoring that spatial task configuration shapes how ASE assistance translates to balance performance.

The two-way intervention × direction interaction revealed that, across every task orientation, ASE use lowered the absolute value of MEAN ML, while the intervention’s main effect showed a parallel and significant reduction in ML APDF10. Taken together, these findings indicate that relieving arm load with ASE support consistently curbed lateral sway—both in its average magnitude and in its fine-scale variability—during the repetitive reaches. This interpretation aligns with recent evidence showing that a passive ASE reduced whole-body postural sway by roughly 18% in static load-holding tasks [14]. Nevertheless, the stabilizing benefit was largely restricted to the ML axis: ASE use produced no statistically significant improvement in MEAN AP or any AP APDF metric, and the corresponding values showed only a slight, non-significant increase. One possible explanation is that balance adjustments in the AP direction were maintained through other compensatory strategies, limiting the observable impact of ASEs. This tendency may tentatively reflect compensatory adjustments in trunk or joint kinematics along the AP axis. Supporting this view, Kuber and Rashedi (2025) [34] found that although an assistive exoskeleton reduced sway in the ML plane, it also prolonged trunk flexion durations and increased AP movement variability, suggesting compensatory adjustments to maintain balance. Likewise, Garcia et al. (2025) [35] reported that exoskeleton use lowered upper-limb muscular demands but was associated with greater dorsiflexion, knee flexion, and thorax tilt—changes that may indicate increased reliance on compensatory strategies in the AP direction—while a reduction in pelvic tilt suggested improved stability in the ML direction. Together, these findings imply that reducing upper-limb load can redistribute postural control across the trunk and lower limbs. However, since such kinematic variables were not directly measured in the present study, this interpretation should be regarded as tentative. Future research that includes quantitative kinematic analyses, such as trunk angles or joint amplitudes, is needed to verify whether ASE use indeed induces compensatory motion patterns in the AP direction.

Task direction emerged as the strongest factor influencing CoP position and variability. During left-facing tasks, participants moved their dominant (right) hand across the body midline, which likely required a weight shift toward the left side of the feet; this produced the largest leftward bias and ML variability. Such cross-midline reaching generally induces asymmetric loading, which can help explain the larger ML deviations observed in these tasks. By contrast, front-facing tasks involved minimal trunk rotation and therefore showed the smallest AP displacement. These patterns mirror previous findings that asymmetric upper-limb actions displace the CoP toward the side on which the task is performed. The intervention × direction interaction for MEAN ML confirmed that ASE use effectively reduced lateral CoP displacement in every task direction, with reductions of up to 20.3%.

Although the two target distances differed by only 3.4 cm (65.5 vs. 68.9 cm)—a small shift within a normal reach envelope—extending to the farther target still produced statistically significant increases in ML APDF50 and ML APDF90, and a numerically lower MEAN AP. These findings indicate that even minor changes in reaching distance can influence sway variability and balance regulation. The accompanying large standard deviation cautions against interpreting the MEAN AP drop as a definitive posterior shift; nevertheless, the consistent rise in the higher-percentile APDF metrics indicates that even a modest extension can broaden the distribution of sway events. In other words, the postural-control system was sensitive to this subtle spatial change, registering measurable—albeit small—adjustments rather than wholesale alterations in balance strategy.

The interaction results show that the benefits of ASE use depend strongly on the direction in which the participant reached. When they reached straight ahead, the devices clearly reduced mediolateral CoP motion. Yet during left- or right-facing reaches, ASE use did not improve—and occasionally slightly increased—AP sway indicators. In other words, the exoskeletons steadied the body in the ML axis, but their effect on front-back balance weakened when the task required a lateral reach. For practical application, planners should therefore look beyond load weight alone and ask, “Will the job involve mainly front-facing motions, or a substantial proportion of lateral reaches?”—because the latter may yield smaller AP-axis gains from these devices.

Several limitations temper the generalization of these findings. The study recruited only healthy, young men and employed a low-load, fixed-rhythm laboratory simulation, which restricts applicability to broader populations (e.g., females, older adults, manual laborers). Prior studies suggest that females exhibit different trunk stabilization strategies and substantially lower absolute upper-limb strength compared with males [35,36,37], while older adults demonstrate reduced steadiness and stability compared with younger groups [25]; such differences may alter the stabilizing effects of ASEs. Manual laborers could also adapt differently due to long-term musculoskeletal conditioning and exposure to irregular, high-load tasks [38]. In addition, because all participants were right-handed, the findings should not be directly extrapolated to other usage conditions. For example, left-handed users may display a reversed mediolateral CoP bias when reaching across the body midline, and tasks performed with the non-dominant hand or with both hands may elicit different balance strategies; although the stabilizing effect of ASEs on lateral sway is likely to remain consistent, future studies should explicitly verify whether these variations in task laterality influence outcomes. Finally, only two passive ASEs (VEX and Airframe) were evaluated, both using spring- or linkage-based torque assistance, and caution is warranted in extrapolating these findings to other exoskeleton types (e.g., powered, hybrid, or back/shoulder-support devices) with different weight distributions, actuation mechanisms, or support locations, as prior studies have shown that balance responses can vary depending on these design characteristics [14,17].

In sum, passive ASEs moderated lateral CoP deviations without materially improving AP stability, and their benefits were modulated by task direction and reach distance. Designers and practitioners should weigh these axis-specific and context-dependent effects when prescribing ASEs for repetitive tasks, tailoring device selection, workspace layout, and user training to maximize balance support while minimizing unintended sway in other planes.

## 5. Conclusions

This study found that passive arm-support exoskeletons reduced body sway in the mediolateral direction during repetitive arm-reaching tasks, as shown by decreased mediolateral CoP displacement and variability. However, they produced no clear improvement in anteroposterior stability. Task direction exerted the strongest influence on postural control, and even minor extensions in reaching distance induced measurable changes in sway distribution. These findings suggest that, while ASEs can enhance lateral postural control, their benefits remain axis- and context-specific and may not generalize across all task configurations.

## Figures and Tables

**Table 1:** MANOVA results for CoP metrics. Bold values denote statistically significant effects (*p* < 0.05). Asterisks indicate effect-size magnitude based on partial eta-squared (η^2^_p_): * small (η^2^_p_ ≥ 0.01), ** medium (η^2^_p_ ≥ 0.06), and *** large (η^2^_p_ ≥ 0.14) [33]. Very small *p*-values are reported as *p* < 0.001, as provided by the statistical analysis software used in this study.

	CoP Metrics								
**Experimental** **Factors**		**MEAN ML**	**MEAN AP**	**ML** **APDF 10**	**ML** **APDF 50**	**ML** **APDF 90**	**AP** **APDF 10**	**AP** **APDF 50**	**AP** **APDF 90**
Intervention	f	1.217	1.765	**5.879**	1.749	1.543	0.204	0.148	2.197
	*p*	0.297	0.173	**0.003**	0.175	0.215	0.815	0.862	0.112
	η^2^_p_	0.006	0.008	0.028 *	0.008	0.007	0.001	0.001	0.011 *
Direction	f	**2162.525**	**698.140**	**13.881**	**360.496**	**256.762**	**142.423**	**39.148**	**18.033**
	*p*	**<0.001**	**<0.001**	**<0.001**	**<0.001**	**<0.001**	**<0.001**	**<0.001**	**<0.001**
	η^2^_p_	0.913 ***	0.772 ***	0.063 **	0.636 ***	0.554 ***	0.408 ***	0.159 ***	0.08 **
Distance	f	0.112	**10.800**	2.121	**6.702**	**5.464**	0.080	2.754	2.118
	*p*	0.738	**0.001**	0.146	**0.010**	**0.020**	0.778	0.098	0.146
	η^2^_p_	0.000	0.025 *	0.005	0.016 *	0.013 *	0.000	0.007	0.005
Intervention × Direction	f	**3.957**	0.321	1.180	0.574	0.528	0.933	0.556	0.404
	*p*	**0.004**	0.864	0.319	0.682	0.715	0.445	0.695	0.806
	η^2^_p_	0.037 *	0.003	0.011 *	0.006	0.005	0.009	0.005	0.004
Intervention × Distance	f	0.040	0.645	1.111	0.044	0.876	0.629	0.329	0.245
	*p*	0.960	0.525	0.330	0.957	0.417	0.534	0.720	0.783
	η^2^_p_	0.000	0.003	0.005	0.000	0.004	0.003	0.002	0.001
Direction × Distance	f	**6.399**	0.768	0.070	0.450	1.362	2.073	**7.758**	**6.416**
	*p*	**0.002**	0.465	0.932	0.638	0.257	0.127	**<0.001**	**0.002**
	η^2^_p_	0.030 *	0.004	0.000	0.002	0.007	0.010 *	0.036 *	0.030 *
Intervention × Direction × Distance	f	0.084	0.278	0.237	0.082	0.396	1.384	0.474	0.610
	*p*	0.987	0.892	0.918	0.988	0.812	0.239	0.755	0.655
	η^2^_p_	0.001	0.003	0.002	0.001	0.004	0.013 *	0.005	0.006

**Table 2:** Mean ± SD comparisons of CoP metrics across intervention conditions. Statistical significance was assessed via MANOVA, with post hoc SNK tests identifying condition-level differences (*p* < 0.05). Superscript letters indicate significant differences. * indicates statistical significance at *p* < 0.05. MEAN ML and MEAN AP are reported in centimeters (one decimal place); normalized APDF values (range 0–1) are reported to three decimal places.

Postural Stability Metrics	Intervention			*p*-Value
	*Without*	*VEX*	*Airframe*	
MEAN ML (cm)	−2.84 (±6.60)	−2.78 (±5.78)	−2.56 (±6.00)	0.297
MEAN AP (cm)	2.32 (±3.10)	2.63 (±3.08)	2.59 (±3.10)	0.173
ML APDF10	0.134 (±0.076) ^A^	0.116 (±0.061) ^B^	0.110 (±0.057) ^B^	0.003 *
ML APDF50	0.488 (±0.150)	0.472 (±0.156)	0.471 (±0.154)	0.175
ML APDF90	0.797 (±0.101)	0.789 (±0.093)	0.784 (±0.106)	0.215
AP APDF10	0.184 (±0.094)	0.186 (±0.094)	0.181 (±0.094)	0.815
AP APDF50	0.449 (±0.106)	0.454 (±0.091)	0.454 (±0.093)	0.862
AP APDF90	0.711 (±0.098)	0.727 (±0.084)	0.732 (±0.090)	0.112

**Table 3:** Mean ± SD comparisons of CoP metrics across task-direction conditions. Statistical significance was assessed via MANOVA, with post hoc SNK tests identifying condition-level differences (*p* < 0.05). Superscript letters indicate significant differences. * indicates statistical significance at *p* < 0.05. MEAN ML and MEAN AP are reported in centimeters (one decimal place); normalized APDF values (range 0–1) are reported to three decimal places. Very small *p*-values are reported as *p* < 0.001, as provided by the statistical analysis software used in this study.

Postural Stability Metrics	Task Direction			*p*-Value
	*Left-Facing*	*Front-Facing*	*Right-Facing*	
MEAN ML (cm)	−9.41 (±1.89) ^A^	−3.61 (±1.37) ^B^	4.79 (±2.25) ^C^	<0.001 *
MEAN AP (cm)	4.38 (±1.30) ^A^	−1.29 (±1.85) ^B^	4.47 (±1.30) ^A^	<0.001 *
ML APDF10	0.142 (±0.070) ^A^	0.113 (±0.073) ^B^	0.105 (±0.044) ^B^	<0.001 *
ML APDF50	0.619 (±0.082) ^A^	0.323 (±0.095) ^B^	0.490 (±0.103) ^C^	<0.001 *
ML APDF90	0.871 (±0.045) ^A^	0.693 (±0.080) ^B^	0.808 (±0.073) ^C^	<0.001 *
AP APDF10	0.210 (±0.081) ^A^	0.102 (±0.061) ^B^	0.240 (±0.076) ^C^	<0.001 *
AP APDF50	0.440 (±0.084) ^A^	0.414 (±0.108) ^B^	0.504 (±0.072) ^C^	<0.001 *
AP APDF90	0.689 (±0.082) ^A^	0.733 (±0.109) ^B^	0.748 (±0.066) ^B^	<0.001 *

**Table 4:** Mean ± SD comparisons of CoP metrics across working-distance conditions. Statistical significance was assessed via MANOVA, with * indicating *p* < 0.05. MEAN ML and MEAN AP are reported in centimeters (one decimal place); normalized APDF values (range 0–1) are reported to three decimal places.

Postural Stability Metrics	Working Distance		*p*-Value
	*Near*	*Far*	
MEAN ML (cm)	−2.78 (±5.82)	−2.68 (±6.43)	0.738
MEAN AP (cm)	2.75 (±2.97)	2.27 (±3.19)	0.001 *
ML APDF10	0.116 (±0.063)	0.124 (±0.068)	0.146
ML APDF50	0.466 (±0.153)	0.488 (±0.153)	0.010 *
ML APDF90	0.783 (±0.105)	0.798 (±0.095)	0.020 *
AP APDF10	0.185 (±0.096)	0.183 (±0.092)	0.778
AP APDF50	0.445 (±0.100)	0.460 (±0.093)	0.098
AP APDF90	0.717 (±0.096)	0.730 (±0.085)	0.146

## Data Availability

All data included in this study are available upon request to the corresponding author.

## Abbreviations

The following abbreviations are used in this manuscript:

AP	Anteroposterior
APDF	Amplitude Probability Density Function
ASE	Arm-Support Exoskeleton
BSE	Back-Support Exoskeleton
CoP	Center of Pressure
EMG	Electromyographic
MANOVA	Multivariate Analysis of Variance
ML	Mediolateral
SNK	Student–Newman–Keuls
